# Attitudes of nurses and physicians towards nurse-physician collaboration in northwest Ethiopia: a hospital based cross-sectional study

**DOI:** 10.1186/s12912-014-0037-7

**Published:** 2014-11-19

**Authors:** Eden Amsalu, Brihanu Boru, Firehiwot Getahun, Begna Tulu

**Affiliations:** Department of Nursing, College of Medicine and Health Sciences, Bahir Dar University, P.O. Box 79, Bahir Dar, Ethiopia; Department of Nursing, College of Medicine and Health Sciences, University of Gonder, Gonder, Ethiopia; Department of Microbilogy, College of Medicine and Health Sciences, Bahir Dar University, P.O. Box 79, Bahir Dar, Ethiopia

**Keywords:** Attitudes, Collaboration, Nurse, Physician, Nurse-physician

## Abstract

**Background:**

Collaboration between professionals is important in health institutions where most activities are team-performed. Ineffective nurse-physician collaboration affects patient outcome, nurses’ job satisfaction and organizational cost and is challenged by personal, interpersonal and organizational factors. The main objective of this study was to assess attitudes of nurses and physicians towards nurse-physician collaboration and the level of satisfaction with regard to quality of collaboration between them at Referral Hospitals of Northwest Ethiopia, from February 1st to April 30, 2013.

**Methods:**

An institution based cross-sectional study was conducted among 176 nurses and 53 physicians working in Felegehiwot and Gondar University Referral Hospitals. Data were collected using self-administered questionnaires. Attitudes of nurses and physicians were measured using Jefferson scale of attitudes towards nurse-physician Collaboration. Results were summarized using descriptive statistics and difference of means and proportions were evaluated using student *t* test p <0.05 was considered as significant.

**Result:**

The overall response rate was 90.50%. Nurses demonstrate more favorable attitudes than physicians with mean score of 49.63 and 47.49 and standard error of mean 0.474 and 0.931 respectively with p = 0.043. For the Jefferson Scale Attitudes towards Nurse-Physician Collaboration includes four subscales, which are: 1) shared education and teamwork, 2) Caring vs curing, 3) nurses autonomy and 4) physician dominance. Nurses scored higher on three subscales (1, 2 and 4). However, statistically significant differences were noted with regard to subscales 2 and 4 (p = 0.01, p = 0.004, respectively).

**Conclusion:**

This study identified that neither nurses nor physicians were satisfied with their current collaboration and nurses demonstrated less satisfaction with the current nurse physician collaboration. As compared with physicians nurses had more favorable attitudes towards collaboration specifically toward nurses’ contributions to the psychosocial and educational aspects of patient care, and stronger rejection of a totally dominant physician role.

## Background

Collaboration implies “collective action toward a common goal in the spirit of trust and harmony”, Makharam 1985 [1]. In the context of healthcare, collaboration is understood as the way in which physicians and nurses interact with each other in relation to clinical decision making. Collaboration involves direct and open communication, respect for different perspectives, and mutual responsibility for problem solving [1,2]. Collaboration between health professionals is essential to achieve clinical outcomes high quality particularly in health institutions where most activities are team-performed [3,4]. Nurse physician collaboration affects patient outcomes [5-9], nurses’ job satisfaction [10], and organizational cost [8,9]. Especially, in the inpatient care unit where patients stay longer away from their home and depend on care and guidance of the health professionals [11-13].

Communication between health care workers accounts for the major part of the information flow in health care, and growing evidence indicates that errors in communication give rise to substantial preventable clinical morbidity and mortality [14-16]. Poor communication leads to misunderstandings, errors, and on-going conflict between nurses and physicians [9,10,17,18]. The Joint Commission on Accreditation of Health care Organizations (JCAHO) reported that communication failures among professionals caused 70% of 2,455 reported sentinel events, with about 75% of the patients dying as a result. The report also underlines negative nurse-physician relationship continues to be one of the sources for increased dissatisfaction amongst physicians, nurses and patients and hampers retention. As a result to improve the effectiveness of communication among caregivers is the second 2013 National Patient Safety goal of the JCAHO [4,9,17].

Findings indicated the nurses’ practice environment and nurse-physician collaboration were strong predictors of job satisfaction [10,16]. Besides effective collaboration poses challenges because of barriers such as gender and class differences, hierarchical organizational structures, and physicians’ belief that they are the final arbiter of clinical decisions [17].

In an effort to improve patient safety, hospitals across the world are turning to outside industries for lessons in communication and quality improvement [14]. It is essential for us to understand the attitudes of nurses and physicians towards collaboration better. Otherwise we can hardly plan whether education or training is needed to foster collaboration.

In Ethiopia, the health coverage is raising from its very limited distribution which was only focused on the urban setup for so many years. Recently, a large number of hospitals, health centers, and health posts are being built in every corner of the country. Especially, the government is working very hard in strengthening the primary health care by assigning more than forty thousand health extension workers throughout the country who are directly working with the community. On the other way, the health care system is suffering from the lack of highly qualified and diversified health professionals, and conducive working environments.

Though there is no published document regarding nurse physician collaboration in Ethiopia, the health system currently exercised indicates nurses are not fully exercising their autonomy while working with physicians and physicians demonstrate total dominant role almost in every step of patient care. This minimizes the contribution of nurses to health care delivery system. This makes us wonder what is going on in their mind about their collaboration and interests us to generate legal evidence towards nurse physician collaboration. Therefore, this study aimed to assess attitudes of nurses and physicians towards nurse physician collaboration.

## Methods

### Aim of the study

- Exploration of the attitudes towards nurse-physician collaboration in nurses and physician, and possible differences between these two groups,
- Exploration of the level of satisfaction among nurses and physicians regarding the quality of collaboration between them, and possible differences between these two groups,
- Exploration of the metric properties of the instrument Jefferson Scale of Attitudes towards nurse-physician collaboration

### Ethical consideration

Ethical approval was obtained from Research Ethical and Review Committee of University of Gondar. Additional permission was obtained from Gondar and Felegehiwot Hospitals medical director offices. The purpose of the study was explained to the participants and informed consent was also obtained before data collection. To keep the confidentiality of the participants, personal identifiers was not included in the data collection format and ensured throughout the research process and the information was utilized only for research purpose. Participation was entirely voluntary.

### Study area, period and population

This institution based cross sectional study was conducted from February to April 2013 G.C in Felegehiwot and Gondar referral hospitals. The hospitals are located 560 km and 740 km northwest of the capital city of Ethiopia, Addis Ababa, respectively. Gondar University hospital is the only referral hospital in Gondar town and has more than 400 inpatient beds and 559 health professionals in it among them 267 are nurses and 129 are physicians. Felegehiwot Hospital is also the only referral hospital in Bair Dar town which accounts for about 400 inpatient beds and 213 health professionals including 31 physicians’ and 143 nurses.

The study population was all nurses and physicians who were working in Felegehiwot & Gondar referral hospitals during the study period. They were selected using stratified random sampling technique.

### Sample size and sampling procedure

The sample size (n) was calculated using the formula to estimate a single population proportion: n = [(Zα/2)^2^ p(1-p)/d^2^]; where, **n** = required sample size, **Z** = critical value for normal distribution at 95% confidence level which equals to 1.96 (z value at α = 0.05), **P** = proportion of persons with favourable attitudes towards nurse physician collaboration, and **d** = margin of error 5%. Using population correction formula since the total target population is less than 10,000, n/1 + (n/N) = 384.2/1 + (384.2/570) = **229.5**. Adding 10% for non-response rate 229.5 + 23 = 252.5. Thus, the required total sample size for the study was **253** in which 182 Nurses and 71 Physicians.

Stratified random sampling technique was used. The total population was stratified by profession to nurses and physicians and sample was taken from each stratum proportionally. Finally, from each hospital, nurses and physicians were allocated proportionally and selected using a simple random method (lottery method) to attain the final individual. The sampling frame was obtained from the lists of nurses and physicians from each ward.

### Operational definition

- **Favorable attitudes towards nurse physician collaboration:** A higher factor score on overall score of Jefferson scale of attitudes towards collaboration.
- **Unfavorable attitudes towards nurse-physician collaboration:** A lower factor score on overall score of Jefferson scale of attitudes towards collaboration.

### Data collection procedure

**Data collector:** Data collection was facilitated by two trained BSc nursing students.

**Instrument:** the attitudes of nurses and physicians was measured by Jefferson Scale of Attitudes toward Nurse-Physician Collaboration. The tool was originally developed by Hojat and Herman in 1985, and was validated in a sample of medical students and acute care nurses. Furthermore, the tool was modified in 2003 by Hojat et al. [19]. This tool was supported by psychometric evidence including construct validity and internal consistency reliability that can be used as a research or evaluative tool in western countries to measure attitudes towards nurse-physician collaboration [20,21].

To check whether it works in Ethiopia, pre-test was conducted in Goba referral Hospital, found in south western part of the country. Inorder to do that about 5% of the total sample size of nurses and physicians were included in the pre-test and finally confirmed that the tool can be applied in Ethiopian context.

The tool includes 15 items, which are grouped in four subscales. These subscales are:

- “Shared education and team work” (7 items), which includes items such as “During their education, medical and nursing students should be involved in teamwork in order to understand their respective roles”
- “Caring as opposed to curing” (3 items), which includes “Nurses have special expertise in patient education and psychological counseling”
- “Nurse's autonomy” (3 items), which includes “Nurses should clarify a physician’s order when they feel that it might have the potential for detrimental effects on the patient” and
- “Physician's dominance” (2 items), including “Doctors should be the dominant authority in all healthcare matters” [20].

Thirteen of the 15 items were judged to be a reflection of a favorable attitudes (items 1 through 13). The responses to these items were directly coded (4 = strongly agree, 3 = agree, 2 = disagree, 1 = strongly disagree). Two items were judged to be a reflection of unfavorable attitudes toward nurse- physician collaboration (Items 14 and 15). These items were reversely scored (recoded: 1 = strongly agree, 2 = agree, 3 = disagree, 4 = strongly disagree). Overall score was calculated by adding each individual’s scores out of 60. After summing the scores, the mean was calculated for each profession.

Additionally, in order to assess the nurse-physician level of satisfaction an item was added which asks nurses and physicians to rate their level of satisfaction.

**Data collection method:** Data were collected by administering written questionnaire to study participants.

### Data quality control

In order to maintain the quality of the data, the questionnaires were checked for completeness before data entry. The data were interred in software called Epi Info version 5.3.3 to point out errors made during data collection automatically then transferred to SPSS. Furthermore, training was given to data facilitators and the overall data collection process was monitored by supervisor.

### Data processing and analysis

Results were summarized using descriptive statistics including frequencies and mean. Difference of mean was evaluated using student *t* test and p <0.05 was considered as significant.

## Results

### Demographic characteristics

From the two referral hospitals of North West Ethiopia 253 health professionals were recruited in the study and 229 (91%) responding. Among them 176 nurses and 53 physicians (77% and 23% of total respondents respectively) participated in the study. One hundred thirty three (58%) were male with mean age of 29.2 (SD ± 5.90 and Range 22–58 years) and 50% and majority of them (66%) had less than 5 years experience (22%) (Table 1).

**Table 1:** **Socio-demographic characteristics of nurses (n = 176) and physicians (n = 53)**

**Characteristics**		**Number of nurses (%)**	**Number of physicians (%)**	**Total no. (%)**
Institution	Felegehiwot Hospital	57 (32)	13 (25)	70 (31)
	Gonder Hospital	119 (68)	40 (76)	159 (69)
Gender	Male	89 (51)	44 (83)	133 (58)
	Female	87 (49)	9 (17)	96 (42)
Age	20-25	15 (26)	12 (22)	27 (25)
	26-30	85 (48)	29 (55)	114 (50)
	31-35	15 (6)	11 (21)	26 (11)
	36-40	20 (11)	0	20 (9)
	> 40	10 (6)	1 (2)	11 (5)
Marital status	Married	92 (52)	22 (42)	114 (50)
	Single	77 (44)	31 (59)	108 (47)
	Divorce	5 (3)	-	5 (2)
	Widowed	2 (1)	-	2 (1)
Level of education	Diploma in Nursing	60 (34)	-	60 (26)
	BSc in Nursing	113 (64)	-	113 (49)
	MSc in Nursing	3 (2)	-	3 (1)
	MD	-	48 (91)	48 (21)
	MD + MSc	-	1 (2)	1 (0.4)
	Specialist	-	4 (8)	4 (1)
Length of services	< 5 years	106 (60)	45 (85)	151 (66)
	5-10 years	43 (24)	7 (13)	50 (22)
	10-15 years	17 (10)	0	17 (7)
	>15 years	10 (6)	1 (2)	11 (5)
Area of work	Medical unit	47 (21)	11 (10)	59 (26)
	Surgical	41 (18)	14 (6)	55 (24)
	Emergency	9 (4)	0	9 (4)
	Operation room	8 (4)	0	8 (4)
	Gynecology/obstetrics	1 (0.4)	13 (6)	14 (6)
	Pediatrics	34 (15)	8 (4)	42 (18)
	Ophthalmology	8 (4)	4 (2)	11 (5)
	OPD	11 (5)	1 (0.4)	12 (5)
	Others	17 (7)	1 (1)	18 (8)

### Instruments’ metric properties

The reliability coefficients of Jefferson scale which is used for measurement of the attitudes of Nurses and Physicians towards nurse physician collaboration for this study was 0.79 for nurses, 0.84 for physicians and 0.78 for combined. Reliability coefficients of these magnitudes are in the acceptable range for attitudes scales.

### Differences in mean values

The *t*-test for the analysis of data revealed significant difference between Nurses and Physicians in attitudes towards nurse – physician collaboration (t = 2.05, p value 0.043) with mean score of 49.63 (standared error of mean (SEM) 0.474) and 47.49 (SEM 0.931) respectively (Table 2). The Jefferson Scale has four subscales; shared education and teamwork, caring vs curing, nurses autonomy and physician dominance. Nurses’ score was higher in the following three subscales, 1, 2, and 4, whilst statistically significant difference was noted only with regard to subscales 2 and 4 (p = 0.01, p = 0.004, respectively). On the other hand physicians score higher than nurses on nurses’ autonomy section though not statistically significant (Table 2).

**Table 2:** **Mean values and differences between them with regard to the subscales of the Jefferson scale of attitudes towards N-P collaboration**

**Jefferson scale**	**Profession**	**Mean**	**SD**	**SEM**	**t**	**df**	**P value**
Shared education scale	Nurse	23.33	3.07	.231	1.36	74	.175
	Physician	22.64	3.70	.509			
Caring vs curing	Nurse	10.28	1.67	.126	3.58	85	.001
	Physician	9.34	1.67	.230			
Nurses autonomy	Nurse	10.15	1.67	.126	1.23	86	.223
	Physician	10.47	1.65	.226			
Physician dominance	Nurse	5.87	1.81	.137	2.90	85	.005
	Physician	5.04	1.83	.251			
Overall attitudes	Nurse	49.63	6.20	0.467	2.05	80	0.043
	Physician	47.49	6.80	0.931			

**Figure 1:**
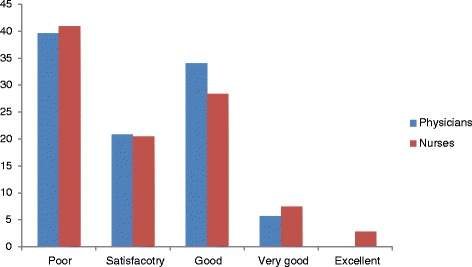
**Level of satisfaction among nurses (n = 176) and physicians (n = 53) regard the quality of collaboration between them.**

The level of satisfaction of nurses and physicians by their collaboration indicated, more than one third of Nurses 72 (41%) as well as physicians 21 (40%) rated as ‘’poor” and 5(3%) nurses and none of the physicians rated as excellent (Figure 1).

## Discussion

It is staggering to note that in this study more than one third of nurses 72(41%) as well as physicians 21(40%) rated their current collaboration as “poor” and only 5(3%) nurses and none of the physicians rated as excellent. This shows that neither nurses nor physicians satisfied with their team work. The same trend was observed by study conducted in Hawassa Teaching Referral Hospital, Ethiopia [22]. However, in the study conducted in Norway the large majority of both nurses (71%) and doctors (79%) considered inter-professional co-operation good at the hospital in which they worked. This might be due to difference in socio-economic status that can affect both individuals and organization [13]. Studies identify that as compared with physicians; more nurses perceive more positively towards collaboration and are less satisfied with the collaboration [17]. The same was true in our case in which Nurses demonstrate significantly more favorable attitudes than Physicians with mean score of 49.63 and 47.49 respectively (P value 0.043). This in line with previous studies conducted in Sweden, Texas, America, and Egypt and cross cultural study including America, Israel, Italian and Mexican [20,23,24]. This could be due to medical training programs that set up a hierarchical model with nurses in a relatively subservient role [10].

Analysis of the Subscales of Jefferson scale reveals nurses scored higher on all subscales except nurses’ autonomy section. Among them caring vs curing (10.28 vs 9.34, p value 0.01) and physician dominance (5.8693 and 5.0377, p value 0.004) were significant factors. This reveals as compared to physicians, nurses in the present study showed more favorable attitudes toward nurses’ contributions to the psychosocial and educational aspects of patient care, and a stronger rejection of a totally dominant physician role. These findings were consistent with previous studies in America, Sweden and Egypt [20,24,25].

There were not enough female physicians in the sample to compare between male and female physicians with regard to positive attitudes toward collaboration. On the other hand there are more female nurses than physicians. This means female nurses has to work more with male physicians. This could be the cause for gender-role-perception based conflicts [26]. However for nurses there was no significant difference. These were in line with a cross cultural study in America, Israel, Italian and Mexican nurses and physicians and studies in Texas and Aurora [19,27].

Neither were there any significant differences in positive attitudes detected in our study between younger and older physicians, and nurses nor in length of service. However, in the study conducted in Texas and Egypt and Aurora experience showed a significant difference [23,27]. This might be due to majority of staff in our study have less than 5 years of experience and the sample of those experienced is not sufficient enough to detect the effect.

Some of the limitations of this study include the design used might establish temporal relationship because of its cross-sectional nature in detecting the favorable attitudes. Similarly, the number of physician participated in the study is smaller than the nurses that may undermine the generalization.

## Conclusion

Generally, this study shows that neither nurses nor physicians were satisfied with their current collaboration. As compared to nurses, physicians were more satisfied with their collaboration with nurses. However nurses had more favorable attitudes towards collaboration than physicians. Specifically, they showed more favorable attitudes toward nurses’ contributions to the psychosocial and educational aspects of patient care, and also showed a stronger rejection of a totally dominant physician role. Thus, the managers of the two hospitals should pay attention on how to create a conducive environment with regard to solve the collaborative activities of their staff and conduct an environmental scan on nurse-physician relationships so that they could plan on-job workshops and seminars on interpersonal and professional communication skills. Finally, further study is proposed to identify factors which affect physician-nurse relationship at large scale by qualitative study.
